# Supplementary material for: Applying the Fokker–Planck equation to grating-based x-ray phase and dark-field imaging
Source: Sci Rep. 2019 Nov 25;9:17465. doi: 10.1038/s41598-019-52283-6 (PMC6877582; doi:10.1038/s41598-019-52283-6)
Supplement: Supplementary file 1 — Supplementary code [file 41598_2019_52283_MOESM1_ESM.pdf]

Supplementary Material

Applying the Fokker-Planck equation to grating-based x-ray phase and dark-field imaging

Kaye S. Morgan, David M. Paganin

Python code to generate an interactive plot for:

1. Equation 16 of the manuscript, modelling a dark-field edge
2. Equation 24 of the manuscript, modelling a phase edge

Also available at: https://github.com/KayeMorgan7/XrayFokkerPlanckSim

```
#!/usr/bin/env python3
# -*- coding: utf-8 -*-
"""
Created on Thu Aug 1 13:11:30 2019

@author: morgank

Built on 'slider_demo.py' from https://matplotlib.org/3.1.1/gallery/widgets/slider_demo.html
"""
import numpy as np
import scipy.special as spy
import matplotlib.pyplot as plt
from matplotlib.widgets import Slider, Button

fig, ax = plt.subplots()
plt.subplots_adjust(left=0.15, bottom=0.5)
x = np.arange(-15.0, 15.0, 0.001)
a0 = 0.25
p0 = 1.5
b0 = 0.5
L0 = 1
R0 = 5
z0=0.5
k0=1.

#Intensity without sample
s = a0*np.sin(x/p0)+b0
l, = plt.plot(x, s, lw=1, color='grey')

#Intensity with sample
ms= b0/2.*(1+spy.erf(x/np.sqrt(2*R0*z0))+(1-spy.erf(x/np.sqrt(2*L0*z0)))) \
    +1/4.*np.complex(0.,1.)*a0*np.exp(-(2*np.complex(0.,1.)*p0*x+(L0+R0)*z0)/(2*p0**2))*\
    (np.exp(L0*z0/(2*p0**2))*(1-np.exp(2*np.complex(0.,1.)*x/p0)*(1+spy.erf((x*p0+np.complex(0.,1.)*R0*z0)/(np.sqrt(2*R0*z0)*p0))) \
    -spy.erf((-x*p0+np.complex(0.,1.)*R0*z0)/(np.sqrt(2*R0*z0)*p0))) \
    +np.exp(R0*z0/(2*p0**2))*((1-spy.erf((x*p0-np.complex(0.,1.)*L0*z0)/(np.sqrt(2*L0*z0)*p0)))  \
    -np.exp(2*np.complex(0.,1.)*x/p0)*(1-spy.erf((x*p0+np.complex(0.,1.)*L0*z0)/(np.sqrt(2*L0*z0)*p0)))))

m, = plt.plot(x, ms, lw=2, color='black')
```

```
plt.xlabel('Position')
plt.ylabel('Intensity')
plt.legend((l,m), ('Without sample', 'With sample'), fontsize='small')
plt.title('Dark field edge')

plt.axis([-15.0, 15.0, 0, 1])

axcolor = 'lightgoldenrodyellow'
axp = plt.axes([0.35, 0.1, 0.55, 0.03], facecolor=axcolor)
axa = plt.axes([0.35, 0.15, 0.55, 0.03], facecolor=axcolor)
axb = plt.axes([0.35,0.2,0.55,0.03], facecolor=axcolor)
axL = plt.axes([0.35,0.25,0.55,0.03], facecolor=axcolor)
axR = plt.axes([0.35,0.3,0.55,0.03], facecolor=axcolor)
axz = plt.axes([0.35,0.35,0.55,0.03], facecolor=axcolor)


sp = Slider(axp, 'Period of illumination p', 0.1, 5.0, valinit=p0)
sa = Slider(axa, 'Amplitude of illumination a', 0.1, 0.5, valinit=a0)
sb = Slider(axb, 'Average intensity b', 0.1, 1.0, valinit=b0)
sL = Slider(axL, 'Diffusion coefficient on left', 0.01, 10, valinit=L0)
sR = Slider(axR, 'Diffusion coefficient on right', 0.01, 10, valinit=R0)
sz = Slider(axz, 'Propagation distance z', 0.01, 1.0, valinit=z0)

def update(val):
    a = sa.val
    p = sp.val
    b = sb.val
    L = sL.val
    R = sR.val
    z = sz.val
    l.set_ydata(a*np.sin(x/p)+b)
    ms=b/2.*(1+spy.erf(x/np.sqrt(2*R*z))+(1-spy.erf(x/np.sqrt(2*L*z)))) \
    +1/4.*np.complex(0.,1.)*a*np.exp(-(2*np.complex(0.,1.)*p*x+(L+R)*z)/(2*p**2))*\
    (np.exp(L*z/(2*p**2))*(1-np.exp(2*np.complex(0.,1.)*x/p)*(1+spy.erf((x*p+np.complex(0.,1.)*R*z)/(np.sqrt(2*R*z)*p))) \
    -spy.erf((-x*p+np.complex(0.,1.)*R*z)/(np.sqrt(2*R*z)*p))) \
    +np.exp(R*z/(2*p**2))*((1-spy.erf((x*p-np.complex(0.,1.)*L*z)/(np.sqrt(2*L*z)*p)))  \
    -np.exp(2*np.complex(0.,1.)*x/p)*(1-spy.erf((x*p+np.complex(0.,1.)*L*z)/(np.sqrt(2*L*z)*p)))))
    m.set_ydata(ms)
    fig.canvas.draw_idle()
```

```
sp.on_changed(update)
sa.on_changed(update)
sb.on_changed(update)
sL.on_changed(update)
sR.on_changed(update)
sz.on_changed(update)

resetax = plt.axes([0.8, 0.025, 0.1, 0.04])
button = Button(resetax, 'Reset', color=axcolor, hovercolor='0.975')


def reset(event):
    sp.reset()
    sa.reset()
    sb.reset()
    sL.reset()
    sR.reset()
    sz.reset()
button.on_clicked(reset)

plt.show()
```

```
#!/usr/bin/env python3
# -*- coding: utf-8 -*-
"""
Created on Thu Aug 1 13:11:30 2019

@author: morgank

Built on 'slider_demo.py' from https://matplotlib.org/3.1.1/gallery/widgets/slider_demo.html
"""
import    numpy as              np
import    matplotlib.pyplot as plt
from matplotlib.widgets import Slider, Button

fig, ax = plt.subplots()
plt.subplots_adjust(left=0.15, bottom=0.5)
x = np.arange(-15.0, 15.0, 0.001)
a0 = 0.25
p0 = 1.5
b0 = 0.5
L0 = 0.3
R0 = 2
z0=0.5
k0=1.

#Intensity without sample
s = a0*np.sin(x/p0)+b0
l, = plt.plot(x, s, lw=1, color='grey')

#Intensity with sample
ms=(z0*(2*np.exp(-x**2)*x*(R0-L0))/(np.sqrt(np.pi)*k0)+1)*(a0*np.sin((x/p0-np.arctan((np.exp(-x**2)*(R0-L0))/(p0*np.sqrt(np.pi)*(k0/z0+(2*np.exp(-x**2)*x*(R0-
L0))/(np.sqrt(np.pi)))))))+b0)
m, = plt.plot(x, ms, lw=2, color='black')
plt.xlabel('Position')
plt.ylabel('Intensity')
plt.legend((l,m), ('Without sample', 'With sample'), fontsize='small')
plt.title('Phase edge')

plt.axis([-15.0, 15.0, 0, 1])
```

```
axcolor = 'lightgoldenrodyellow'
axp = plt.axes([0.35, 0.1, 0.55, 0.03], facecolor=axcolor)
axa = plt.axes([0.35, 0.15, 0.55, 0.03], facecolor=axcolor)
axb = plt.axes([0.35,0.2,0.55,0.03], facecolor=axcolor)
axL = plt.axes([0.35,0.25,0.55,0.03], facecolor=axcolor)
axR = plt.axes([0.35,0.3,0.55,0.03], facecolor=axcolor)
axz = plt.axes([0.35,0.35,0.55,0.03], facecolor=axcolor)


sp = Slider(axp, 'Period of illumination p', 0.1, 5.0, valinit=p0)
sa = Slider(axa, 'Amplitude of illumination a', 0.1, 0.5, valinit=a0)
sb = Slider(axb, 'Average intensity b', 0.1, 1.0, valinit=b0)
sL = Slider(axL, 'Phase shift on left', 0.01, 4, valinit=L0)
sR = Slider(axR, 'Phase shift on right', 0.01, 4, valinit=R0)
sz = Slider(axz, 'Propagation distance z', 0.01, 1.0, valinit=z0)

def update(val):
    a = sa.val
    p = sp.val
    b = sb.val
    L = sL.val
    R = sR.val
    z = sz.val
    l.set_ydata(a*np.sin(x/p)+b)
    ms=(z*(2*np.exp(-x**2)*x*(R-L))/(np.sqrt(np.pi)*k0)+1)*(a*np.sin((x/p-np.arctan((np.exp(-x**2)*(R-L))/(p*np.sqrt(np.pi)*(k0/z+(2*np.exp(-x**2)*x*(R-
L))/(np.sqrt(np.pi)))))))+b)
    m.set_ydata(ms)

    fig.canvas.draw_idle()
sp.on_changed(update)
sa.on_changed(update)
sb.on_changed(update)
sL.on_changed(update)
sR.on_changed(update)
sz.on_changed(update)

resetax = plt.axes([0.8, 0.025, 0.1, 0.04])
button = Button(resetax, 'Reset', color=axcolor, hovercolor='0.975')
```

```
def reset(event):
    sp.reset()
    sa.reset()
    sb.reset()
    sL.reset()
    sR.reset()
    sz.reset()
button.on_clicked(reset)

plt.show()
```
